# Unveiling the Immune Microenvironment’s Role in Breast Cancer: A Glimpse into Promising Frontiers

**DOI:** 10.3390/ijms242015332

**Published:** 2023-10-18

**Authors:** Amalia Kotsifaki, Nektarios Alevizopoulos, Vassiliki Dimopoulou, Athanasios Armakolas

**Affiliations:** Physiology Laboratory, Medical School, National and Kapodistrian University of Athens, 11527 Athens, Greece; amaliakotsifaki96@gmail.com (A.K.); nalevizopoulos@gmail.com (N.A.); vdimopoulou14@gmail.com (V.D.)

**Keywords:** breast cancer, tumor microenvironment, tumor-infiltrating lymphocytes, immune checkpoint inhibitors, tumor-associated macrophages, immunotherapy

## Abstract

Breast cancer (BC), one of the most widespread and devastating diseases affecting women worldwide, presents a significant public health challenge. This review explores the emerging frontiers of research focused on deciphering the intricate interplay between BC cells and the immune microenvironment. Understanding the role of the immune system in BC is critical as it holds promise for novel therapeutic approaches and precision medicine strategies. This review delves into the current literature regarding the immune microenvironment’s contribution to BC initiation, progression, and metastasis. It examines the complex mechanisms by which BC cells interact with various immune cell populations, including tumor-infiltrating lymphocytes (TILs) and tumor-associated macrophages (TAMs). Furthermore, this review highlights the impact of immune-related factors, such as cytokines and immune checkpoint molecules. Additionally, this comprehensive analysis sheds light on the potential biomarkers associated with the immune response in BC, enabling early diagnosis and prognostic assessment. The therapeutic implications of targeting the immune microenvironment are also explored, encompassing immunotherapeutic strategies and combination therapies to enhance treatment efficacy. The significance of this review lies in its potential to pave the way for novel therapeutic interventions, providing clinicians and researchers with essential knowledge to design targeted and personalized treatment regimens for BC patients.

## 1. Introduction

Breast cancer (BC), a heterogeneous and complex disease, currently ranks as the second leading cause of cancer-related deaths worldwide and continues to be the most common malignancy in women [1]. It presents a considerable mental, physical, and socioeconomic burden on families, individuals, and systems of healthcare [2]. Based on molecular characteristics and immunohistochemical (IHC) expression of hormone receptors (HR), BC is commonly grouped into four subtypes: luminal A (ER+, PR+, Ki67 < 20%), luminal B (ER+, PR+ or PR- HER2+ or HER2-, Ki67 > 20%), human epidermal growth factor receptor 2 (HER2)-overexpression (ER+, PR+), and triple-negative BC (TNBC)/basal-like. TNBC does not express progesterone receptor (PR), estrogen receptor (ER), or HER2 on its cell surface (ER-, PR-, HER2-) [3,4,5]. The protein Ki67 is essential for cell growth and is also frequently employed as a marker to evaluate the growth fraction or rate of proliferation of tumor cells [5]. Each of the aforementioned subtypes has unique biological characteristics, clinical behaviors, and treatment responses, highlighting the need for personalized approaches in BC management [6,7].

According to the current literature, traditional BC classification divides the disease into three subtypes based on the expression of the HR and the HER2: HR-positive (HR+)/HER2-negative (HER2-) BC, HER2+ BC, and HR-/HER2- or TNBC [8]. They comprise approximately 70%, 15–20%, and 10–15% of all BC diagnoses, respectively [9]. Approximately 70–75% of invasive BCs exhibit considerably high ER expression, making the ER a key diagnostic factor [10]. More than 50% of individuals with ER-positive BC express PR, while ER-negative BC patients hardly ever do [11,12]. HR/PR+ patients can be successfully treated with ER antagonists like tamoxifen or aromatase inhibitors, while HER2+ BC patients receive treatment with antibodies or other compounds targeting the HER2 pathways, such as trastuzumab, pertuzumab, and lapatinib [13]. Moreover, the elimination of effective therapeutic targets (PR, ER, and HER2) due to the lack of expression in TNBC is associated with adverse effects on diagnosis and treatment, making TNBC the most aggressive BC subtype, highly metastatic, and with poor overall survival rates in about 15% of all BC cases [3]. Since hormone therapy or HER2-targeted medications are ineffective for TNBC patients, chemotherapy and surgery, both of which can be extremely invasive, remain their primary treatment options [14]. However, it is important to note that ongoing efforts are increasingly focused on identifying novel targets for personalized treatment approaches. Recent research underscores the dynamic landscape of TNBC treatment, highlighting the continuous pursuit of innovative strategies to enhance patient outcomes [15,16]. Regarding the metabolism in BC, BC cells modify their nutrient metabolism to adjust to the tumor’s acquiring energy and growth, as metabolic reprogramming is seen as a sign of tumor development [17,18,19].

As research advances, it becomes increasingly evident that the interplay between BC and the immune system is a captivating and complex relationship, brimming with untapped potential. Although BC patients’ outcomes have improved because of alternative options for treatment, numerous individuals continue to develop metastatic disease, a condition that is still exceedingly difficult to treat [17,20]. Current research indicates that BC apart from neoplastic cells also includes the tumor microenvironment (TME), which comprises several cell types, including immune, stromal, and endothelial cells [17]. These cells, making up the TME, interact complexly with cancer cells influencing the microenvironment [21].

Extensive research has shed light on the complex interplay between cancer cells and the immune system within the TME. This evolving field of study has revealed crucial insights into the role of the immune system in shaping tumor progression, treatment response, and patient outcomes [22]. Understanding the immune microenvironment in BC is paramount as it holds the potential to revolutionize diagnosis, prognosis, and therapeutic approaches. There are multiple heterogenous populations in the BC’s immune microenvironment, including immunology cells (lymphocytes, macrophages, NK cells, dendritic cells (DCs), myeloid-derived suppressor cells (MDSCs), etc.) and other immune-mediated factors (cytokines, chemokines) [17,23]. These cells and immune factors have a significant impact on the malignant biological activity of cancer cells through persistent inflammation accelerating the development of BC [24,25]. In addition to influencing tumor immunogenicity, the immune system also plays a dual role in preventing tumor development by activating innate and adaptive immune systems [26]. Deciphering these intricate interactions is crucial for developing novel immunotherapeutic strategies, improving existing treatments, and identifying reliable biomarkers for patient stratification [27].

Surgery combined with chemotherapy continues to be the first-line treatment for BC, with a generally good prognosis [28]. However, several immunotherapy approaches were developed and have been utilized for medical purposes over the past few decades according to immune evasion mechanisms [29]. Scientists have begun taking advantage of the immune response to BC and are developing immunotherapies to carry out a variety of functions, such as preventing BC through vaccination, strengthening the immune system for combating BC, or lowering the mortality rate from female BC when combined with chemotherapy [24]. It effectively conveys the distinction between conventional chemotherapy and immunotherapy, emphasizing the immune system’s role in targeting cancer cells and its potential for fewer adverse outcomes [25].

Immunotherapy, which harnesses the body’s immune system to combat cancer, is now a critical component of certain cancer therapies, which highlights its significance [30]. For instance, immune checkpoint proteins such as CTLA-4, PD-1, and PD-L1 have been identified as targets that prevent the immune system from attacking tumors [31]. More specifically, in lung cancer and melanoma, checkpoint inhibitor medicines reawaken the T cells, resulting in robust responses and long-term survival [32]. Several of them have demonstrated promising clinical effectiveness, including cancer vaccines (CVs), adoptive cell treatments, monoclonal antibodies, cytokine therapies, oncolytic viruses, and inhibitors targeting immune checkpoints. While certain immunotherapeutic strategies have received approval for the treatment of particular cancers, others are still in the preclinical and clinical trial stages [33,34]. Moreover, recent advances in the development and use of novel technologies, such as single-cell RNA sequencing and mass cytometry, have assisted in the delineation of the immune cell landscape within TME at the single-cell level and facilitated the observation and identification of novel subsets of tumor-associated immune cells [25,35,36].

The TME’s involvement in the neoadjuvant setting is currently beginning to emerge [23,32]. This comprehensive review aims to explore the immune microenvironment in BC, focusing on its impact on disease progression and treatment response. It examines the multifaceted role of immune cells, the dynamic changes in the immune landscape, and their implications for patient outcomes. Emerging immunotherapeutic approaches, such as immune checkpoint inhibitors (CIs), chimeric antigen receptor T cell (CAR-T cell) therapy, and tumor-infiltrating lymphocytes (TILs) therapy, encounter challenges in targeting BC effectively. In addition to biomarker-based immunotherapy, other important biomarkers have been considered in the context of BC. In this regard, the Receptor for Activated C Kinase 1 (RACK1) has been pinpointed as an important biomarker for BC, since its expression directly correlates with overall survival and cancer hallmarks, and it holds high potential as a prognostic value [37,38]. In addition, its importance is also highlighted by its functions at the cancer/immune edge with central implications for the tumor microenvironment [39]. This review also highlights current knowledge gaps, proposes future research directions in this field, and provides a comprehensive overview of the immune microenvironment in BC, emphasizing its crucial role in tumor biology, treatment response, and patient prognosis (Table 1). By elucidating the interactions between cancer cells and the immune system, this review contributes to the growing body of knowledge that will shape the future of immunotherapy for BC.

## 2. Decoding the Multifaceted Roles of Tumor-Infiltrating Lymphocytes (TILs) in BC

### 2.1. TILs Subgroups and Frequency

TILs are commonly referred to as the total of all mononuclear cells in the tumor that are distinguishable by conventional morphological criteria, comprising lymphocytes and plasma cells. Their presence in the tumor is thought to indicate the presence of a continuing immune response directed against the tumor by the host [40]. Based on their position inside the TME, these cell types can be further divided into the following categories: the intra-epithelial compartment is where stromal TILs (sTILs) and TILs with direct cell-to-cell contact with cancer cells are found [41]. In addition to B and T cells, stromal TILs include plasma cells while excluding polymorphs. These specific immune cell components, such as plasma cells, play a crucial role in organizing the immune response within the TME by secreting essential cytokines. Therefore, stromal TILs encompass a range of lymphocytes, including B cells, T cells, and plasma cells, contributing to the intricate immune modulation in the TME [42,43,44]. TILs can be further categorized into various cell types, including CD8+ cytotoxic T lymphocytes, CD8+ tissue-resident memory T (TRM) cells, natural killer (NK) cells, CD4+ T helper 1/2/17 (Th1, Th2, and Th17) cells, CD4+ regulatory T cells (Tregs), CD4+ follicular helper (Tfh) T cells, and tumor-infiltrating B cells (Table 2) [42]. Their composition can change during BC progression and treatment. Tregs are defined by the expression of Forkhead Box P3 protein (FOXP3) in the nucleus as well as CD25 and CTLA-4 on the cell surface [45].

In BC, higher levels of CD8+ TILs have been associated with improved clinical outcomes, including better overall survival and disease-free survival [46]. Furthermore, the presence of Tregs within the TME has been associated with a poor prognosis and reduced survival outcomes [8,47]. Tregs can inhibit the function of effector T cells, including cytotoxic T cells, thereby dampening the anti-tumor immune response. Higher Treg infiltration is associated with immunosuppression and immune evasion mechanisms in BC [48].

In TME, the density and functional status of NK cells within the TME have been linked to clinical outcomes. Higher infiltration of activated NK cells has been correlated with better prognosis, including improved overall survival and disease-free survival [49,50]. B cells can play dual roles in the anti-tumor immune response. On one hand, they can produce tumor-targeting antibodies and induce antibody-dependent cellular cytotoxicity (ADCC) mediated by other immune cells. On the other hand, B cells can promote tumor progression through the production of immunosuppressive cytokines and interactions with other immune cells [8,51]. A subpopulation of B cells called regulatory B (Breg) cells encourages B cell development into Breg cells, detects soluble programmed death-ligand 1 (PD-L1), and activates naive CD4+ T cells to differentiate into Treg cell subtypes [47].

The frequency of TILs can vary widely among BC subtypes. Typically, TNBC and HER2+ BC have higher TIL levels compared to HR+ BCs. TNBC often exhibits a more pronounced TIL infiltration [52]. They are quantified using histopathological assessment of tumor tissue sections, primarily through the evaluation of hematoxylin and eosin (H&E)-stained slides [53]. In some BCs, there is a shift from an inflammatory TIL profile dominated by cytotoxic T cells to a more regulatory TIL profile with a higher proportion of Tregs. This shift can inhibit the anti-tumor immune response and promote immune evasion. In some cases, TILs are organized into tertiary lymphoid structures (TLS) within the TME [54]. TLS can contain B cells, follicular dendritic cells, and high endothelial venules, resembling lymphoid structures found in secondary lymphoid organs. The presence of TLS is associated with a better prognosis in BC [55].

### 2.2. Distribution, Density, and Functional Characteristics within the Tumor

Numerous studies have examined the distribution, density, and functional characteristics of TILs and their significance in the prognosis and therapy of BC. TILs are distributed and densely packed throughout the tumor stroma (TS), invasive tumor nests (TNs), and the surrounding regions. Variable BC subtypes can have variable TIL distributions and densities [40]. For instance, relative to other subtypes, TNBC often has greater numbers of TILs. TILs may be distributed unevenly within the tumor, often exhibiting a higher density towards the invasive tumor front [56].

TILs can have different spatial distribution patterns within the tumor. They may be located at the tumor periphery (margin), within the tumor (intratumoral), or diffusely infiltrating throughout the tumor stroma. Intratumoral TILs are often associated with a more aggressive tumor phenotype [57]. Moreover, BCs can exhibit intratumoral heterogeneity in TIL distribution, with some regions being highly infiltrated while others are devoid of TILs. This heterogeneity can impact treatment response and prognosis assessment [58].

The presence of abundant TILs in the tumor microenvironment classifies a tumor as “hot”, indicating an active anti-tumor immune response. Conversely, “cold” tumors lack significant TIL infiltration and are less responsive to immunotherapy. “Hot” tumors often have tertiary lymphoid structures (TLSs) within them. Several studies have reported that increased TIL levels are associated with better overall survival, disease-free survival, and therapeutic responsiveness, particularly in TNBC and HER2+ BC [8,59].

Early histological examinations of tumor samples had already revealed variable TIL distribution across tumor types and the presence of various immune cell subsets both inside and outside the tumor [60]. In particular, it was discovered that the distribution of TILs was highly ordered in some places rather than being random. NK cells are predominantly found in the stroma, whereas B cells are primarily situated at the invasive margin and often clustered inside TLSs adjacent to the tumor [42]. Other T cells that are reactive to tumors, such as CD8+ cytotoxic T cells, are essential for the immune surveillance and eradication of tumors [61]. TILs with an activated phenotype, characterized by the expression of immune checkpoint molecules like PD-1 and CTLA-4, exhibit better clinical performance and are more responsive to immune checkpoint inhibitors [8]. According to this evidence, TILs’ functional characteristics, including their level of activation and cytokine production, can shed light on the tumor immune milieu [51,52].

### 2.3. Investigating the Prognostic Value of TILs in Breast Cancer

Multiple studies have demonstrated a link between the density and presence of TILs in BC and increased overall survival rates [8]. Some investigations apply single-cell RNA-sequencing analysis to demonstrate TILs’ prognostic value [62,63]. This association has been observed across the different BC subtypes. A recent meta-analysis by Gao et al. reveals that in BC patients with TNBC and HER2-enriched molecular subtypes, increased TILs are associated with favorable survival outcomes and predict pathologic complete response (pCR) [64]. Another critical meta-analysis highlighted that TNBC and HER2+ BC patients who had higher levels of TILs presented higher rates of response to neoadjuvant chemotherapy and better prognoses, but those who had HR+ BC did not [65]. It is unclear whether TILs behave differently in diverse molecular subtypes of BC [66]. Their prognostic and predictive utility in various molecular subtypes of BC is also still debatable [40].

Increased TILs were shown to positively correlate with neoadjuvant treatment response and clinical benefit in early HER2+ BC, similar to what was seen in TNBC, according to several studies evaluating the role of TILs in this cancer [8,59,67]. TNBC is believed to be the most immunogenic subtype of cancer due to its high TIL levels [68]. Consequently, TNBC shows a significant infiltration of Treg cells, indicating its strong immunogenicity [3,47]. However, other researchers think that Treg cells improve the prognosis for BC patients by producing IL-10, which inhibits the immune system’s T cells. A study by Li et al. found that TNBC patients had the highest percentages of Breg cells and PD-1+ Breg cells among various BC subtypes, suggesting a worse prognosis for TNBC compared to other BC subtypes [47]. Moreover, TIL enrichment can identify immune-vulnerable and highly immunogenic BCs. It may be advantageous for individuals with better prognoses, who could benefit from less intensive therapies, as higher TIL levels reduce disease recurrence and prolong disease-free intervals [60]. This effect has been observed in different BC subtypes, including HER2+ and TNBC [3,69,70]. In HER2+ malignancies, high TIL levels have been linked to altered chemosensitivity and increased adjuvant trastuzumab therapy efficacy [71]. Regarding how lymphocyte infiltration impacts prognoses and therapy prediction in patients with ER+/HER2 malignancies, there is, however, a paucity of data [72].

Additionally, the prognostic value of TILs in BC can vary in the context of other established prognostic factors. TILs also offer independent prognostic information beyond traditional factors such as tumor size, lymph node involvement, and histological grade. For example, studies have shown that the prognostic impact of TILs may be more pronounced in HER2+ and TNBC subtypes compared to HR+ tumors [40,73]. Interestingly, while TILs have shown a predictive advantage in ovarian cancer and BC, this advantage doesn’t always result in a therapeutic benefit when it comes to immune checkpoint blockade therapy for these types of cancer. This suggests that there may be variations in the quality of the TIL response [42]. High densities of CD8+ TILs have consistently been associated with better clinical outcomes, including improved overall survival and disease-free survival. However, some studies have additionally emphasized the significance and prognostic relevance of stromal TILs [74]. Higher stromal TIL levels are linked to a better outcome in HER2+ BC patients [43].

High stromal tumor necrosis factor (TNF) levels correlate with improved prognosis and enhanced response to trastuzumab and chemotherapy [8]. High TILs at baseline were found to be a reliable indicator of pCR response to neoadjuvant chemotherapy and trastuzumab in two significant meta-analyses [75,76]. The least immunogenic subtype of BC is HR+/HER2- BC, which has a lower mean TIL count and lower tumor mutational burden. This might be brought on by increased ER expression, increased Th2 infiltration, decreased MHC-II expression, suppression of interferon-signaling, and diminished CD8+ T cell cytolytic activity [77,78]. The considerable heterogeneity of HR+/HER2+ BCs may warrant additional research into the inverse prognostic significance of TILs in this subgroup of BC. While FOXP3+ cells, which comprise CD4+/FOXP3+ cells (Treg) and CD8+/FOXP3+ cells (activated CD8 T cells), are in agreement, high CD8+ TIL infiltration is related to a decrease in BC-specific survival [79]. In HR+/HER2- BCs lacking CD8+ TILs, high numbers of FOXP3+ TILs are related to poor survival [80]. It is important to note that although many studies have consistently reported associations between TILs and BC prognosis, variations in assessment methodologies and cutoff values exist. Standardization of assessment methods and further research is necessary to fully understand the prognostic value of TILs in BC and integrate them into clinical practice [8,43].

## 3. The Multifaceted Roles of Tumor-Associated Macrophages (TAMs) in Breast Cancer

### 3.1. The Presence of TAMs in the Breast Cancer Microenvironment

Among the various immune cells found in the BC microenvironment, tumor-associated macrophages (TAMs) have emerged as a significant focus of research. TAMs, a diverse population of infiltrating macrophages, have been demonstrated to influence tumor growth, angiogenesis, invasion, and metastasis [81]. TAMs are typically quantified using IHC or immunofluorescence staining on tumor tissue sections. They are often reported as the percentage of TAMs relative to the total TS area [82].

This significant part of the TME demonstrates remarkable phenotypic flexibility in its influence on tumor development [83]. TAMs are recruited to the tumor site through chemotactic signals released by cancer cells and stromal cells. The tumor’s inflammatory milieu and secretion of cytokines, such as CSF-1 and CCL2 (also known as monocyte chemoattractant protein-1), contribute to the attraction and polarization of TAMs into distinct subtypes [81,84]. Their abundance in the BC microenvironment is associated with poor clinical outcomes, indicating their detrimental effects on disease progression [81,85].

BC exhibits intratumor heterogeneity, which is crucial for determining its prognosis and treatment effectiveness [81]. Some TAMs may express both M1 and M2 markers, and their specific roles within the TME can vary. The TME can reprogram monocytes and macrophages toward an M2-like phenotype through cytokines, such as IL-4, IL-10, and TGF-β. This polarization can inhibit anti-tumor immune responses and promote immunosuppression [86,87]. TAMs are often found in perivascular areas within the tumor, where they play a role in angiogenesis and support tumor vasculature. They can also accumulate in hypoxic regions, where their presence is associated with tumor aggressiveness [87].

Furthermore, they can be distributed throughout the tumor, including the tumor margin (invasive front) and the tumor core. The distribution pattern may vary depending on the BC subtype [88]. High TAM density at the invasive front is often associated with increased invasiveness and metastatic potential [89]. Some BCs exhibit TAM gradients, with higher TAM densities at the tumor periphery and lower densities at the tumor center [87]. TAMs can also be present in tumor-draining lymph nodes, where they may contribute to lymph node metastasis [90].

To define intratumor morphological and functional heterogeneity, methods like the separation of TS and TN can be used [91]. By analyzing these components separately, researchers can gain insights into the diverse cellular and molecular interactions within the tumor, providing valuable information about tumor growth, invasion, and the potential influence of the microenvironment on cancer progression [92]. Macrophage infiltration in TN is described as tumor-infiltrating macrophages within epithelial cancer cells [93]. These specialized immune cells play a crucial role in the TME, impacting cancer progression and therapeutic responses. When macrophages infiltrate the TN, they can interact with cancer cells directly, influencing their behavior and promoting tumor growth [94]. The interactions between tumor cells and macrophages within the TN can contribute to the development of an immunosuppressive microenvironment and facilitate immune evasion by cancer cells [95]. 

In addition, the morphology of cancer cells can be used to identify five intratumor morphological features: tubular, alveolar, solid, and trabecular structures [93]. For example, TNBC is characterized by low intratumor heterogeneity and often exhibits a single dominant morphological structure [96]. BCs with alveolar and trabecular characteristics often exhibit a worse neoadjuvant chemotherapy response, lower metastasis-free survival, and higher risk of lymph node and distant metastasis [97]. These morphological structures have different macrophage distributions and expression of genes for SI-CLP, CD206, and Stabilin-1 [81]. Moreover, the degree of stromal–parenchymal interactions is the basis for another type of heterogeneity in BC. Areas with soft fibrous stroma, coarse fibrous stroma, greatest stromal and parenchymal relationship, parenchymal elements, and gaps of ductal tumor structures are five distinct morphological compartments in human BC [98]. The clinical significance of TAM infiltration in a specific compartment or genetic subtype of BC varies depending on the patient [99].

Regarding TAMs’ impact on the TME’s metabolic profile, it is worth noting that the regulation of metabolic activities and metabolites can influence tumor development. In response to lactic acid produced by cancer cell glycolysis, a significant number of macrophages gather in hypoxic tumor areas and polarize into the M2 phenotype [95]. More specifically, the activation of the Notch pathway by lactate-activated macrophages stimulates the release of chemokine C-C motif ligand 5 (CCL5) [99], which plays a crucial role in mediating metabolic communication between TAMs and breast malignancies [94]. Monoclonal antibodies that block the CCL5–CCR5 axis disrupt the glycolytic metabolic cycle and inhibit cancer cell metastasis [95].

### 3.2. TAMs Subtypes and Their Effect on Tumor Progression, Invasion, and Metastasis

**Figure 1:**
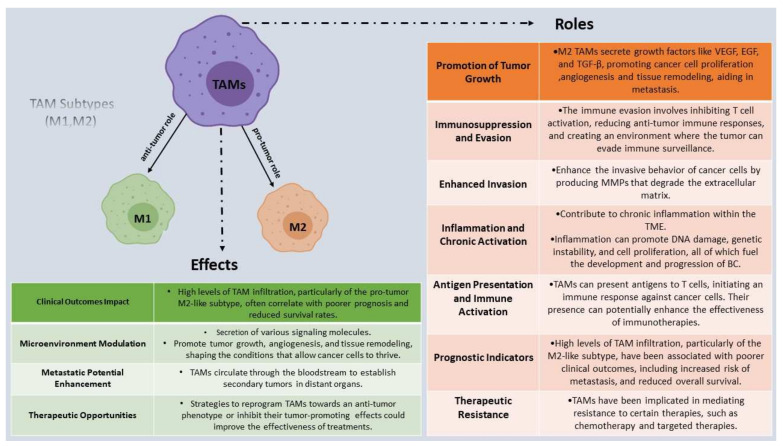
“The Subtypes, Roles, and Effects of Tumor-Associated Macrophages in the Breast Cancer Microenvironment”. This figure provides a detailed overview of the subtypes, roles, and effects of TAMs within the BC microenvironment, highlighting their significant impact on tumor progression and therapeutic outcomes. TME: tumor microenvironment; MMPs: matrix metalloproteinases; BC: breast cancer; TAMs: tumor-associated macrophages.

Macrophages are a diverse cell population that has evolved into two functionally separate subtypes that produce either classically activated M1 macrophages or activated M2 macrophages in response to various environmental factor-based stimuli, respectively (Figure 1) [95,100]. To start with, M1 macrophages often destroy tumors by recognizing and phagocytosing cancer cells [99]. They are activated in response to pro-inflammatory signals and play a role in initiating and promoting anti-tumor immune responses [87]. M1-like TAMs secrete pro-inflammatory cytokines, such as interleukin-12 (IL-12), IL-1β, IL-6, and IL-8, tumor necrosis factor-alpha (TNF-alpha), as well as reactive oxygen species (ROS) and nitric oxide (NO). These factors contribute to tumor cell killing and immune activation [94]. In BC, M1-like TAMs can stimulate the recruitment and activation of cytotoxic T cells and NK cells, enhancing the anti-tumor immune response. In a TME dominated by M1-like TAMs, BC progression is hindered due to their anti-tumorigenic activities [95].

On the contrary, M2 macrophages become more numerous and the predominant TAM type in the TME as the tumor progresses [87]. By encouraging malignant cell dissemination and invasion, metastasis, angiogenesis, cancer stemness, regulating energy metabolism, and immune system evasion, M2 macrophages are commonly referred to as “tumor promotors” that enhance the growth of BC [95]. Patients’ prognoses and treatment outcomes have been demonstrated to worsen when TAM-based infiltration occurs within the primary tumor. Furthermore, they are induced by anti-inflammatory signals and produce immunosuppressive cytokines, such as IL-10 and transforming growth factor-beta (TGF-β), as well as factors that promote angiogenesis and tissue remodeling, such as vascular endothelial growth factor (VEGF) and matrix metalloproteinases (MMPs) [62,101]. Regarding the effect on BC, M2-like TAMs contribute to BC progression and metastasis by promoting immune suppression. They inhibit the activity of cytotoxic T cells and NK cells, impairing the anti-tumor immune response and allowing cancer cells to evade destruction [101]. Additionally, M2-like TAMs support tumor angiogenesis, facilitating the development of new blood vessels to supply nutrients and oxygen to the growing tumor. They also contribute to tissue remodeling, developing a supportive environment for cancer cell invasion and dissemination [102].

Consequently, the balance between M1-like and M2-like TAMs in the TME is critical for BC progression. The phenotypic range of TAMs is considerably more nuanced than previously believed [36]. TAM subsets that exert pro-angiogenic capacities through the expression of pro-angiogenic factors and vascular promotion or favor the formation of pre-metastatic niches in BC have been identified. TAMs within the same cells can express a combination of genes with both M1-like and M2-like signatures that correlate along a similar activation trajectory [103]. An abundance of M2-like TAMs is often associated with poor clinical outcomes, increased tumor growth, invasion, and metastasis [104]. On the other hand, a higher proportion of M1-like TAMs may have a protective effect against cancer progression by enhancing anti-tumor immune responses and inhibiting tumor cell proliferation [93].

### 3.3. Therapeutic Strategies Targeting TAMs in Breast Cancer

Based on the existing knowledge, TAMs are prevalent and constitute approximately between 30 and 50% of the stromal cells in the TME [104]. In advanced malignancy, TAMs have an immunosuppressive M2-like phenotype that is essential for tumor growth, invasion, and migration as well as angiogenesis and immunosuppression [104]. As a result, TAM-targeting medicines are particularly important for anti-cancer approaches (Figure 1). TAMs are predicted to outperform conventional tumor-associated treatments when used as anti-cancer targets, and this will have a positive therapeutic impact. However, targeting TAMs can be challenging and unclear due to the diversity of TAM subtypes [105].

The key objectives of TAM-targeted treatment are to prevent TAM recruitment, deplete TAMs, and reverse TAM polarization [105]. The recruitment of TAMs can be inhibited in part by blocking the effects of chemokines, and most current research focuses on CCL2 and its receptor CCR2 [106]. Firstly, inhibiting the CCL2–CCR2 axis can lessen bone marrow mononuclear cell mobilization and reduce breast macrophage infiltration. According to research, trabectedin and bortezomib can prevent macrophage recruitment by lowering the plasma concentration of CCL2 [107]. Moreover, by enlisting macrophages in recurrent tumors, CCL5 can promote the recurrence of BC. Adjuvant chemotherapy and TNBC recurrence prevention may increasingly focus on CCL5 [108]. The direction of TAMs’ polarization can be controlled by cytokines [105]. For instance, TAMs often become polarized as M1 type when exposed to cytokines generated by CD4+Th1 cells (such as TNF, IL-12, etc.) [109]. An essential mechanism that controls the production of CD4+Th1 cytokines is the NF-kB pathway. The progression of TNBC can be slowed down by activating the NF-kB pathway, which can encourage the polarization of TAMs to the M1 type [110].

In addition, TAM depletion has made extensive use of macrophage apoptosis inducers based on bisphosphonates [109]. Through endocytosis, bisphosphonates are quickly taken up by macrophages [111,112]. By restricting the prenylation of RAS-related proteins, the internalized bisphosphonate can reduce farnesyl diphosphate (FPP) synthase activity and trigger macrophage death [105]. Numerous clinical studies have demonstrated a considerable advantage of bisphosphonate treatment for post-menopausal women with BC. However, it does not apply to women who are menopausal [113,114,115].

The development of TAM and the development of TNBC tumors are both correlated with CSF1 and CCL2. In vivo studies have shown that CSF1 can inhibit the infiltration of TAMs and the development and progression of tumors [116]. In the co-culture system, blocking CSF-1 can have an impact on cancer cells’ ability to produce osteoclasts. Similarly, suppressing CCL2 can prevent M2 recruitment and tumor stem cell renewal, slowing the development of TNBC [105]. Another possible therapeutic approach is to control the expression of PD-1/PD-L1 by controlling the different cytokines released by TAMs. For instance, the JAK/STAT3 signal is connected to the IFN-induced overexpression of PD-L1 [89]. Therefore, it makes therapeutic sense to combine TGF-inhibitors with anti-PD-1/PD-L1 specific antibodies, and corresponding clinical trials are also under progress [117].

Drugs, metals, and microRNAs (miRNAs) may all be carried by nanoparticles, and they can all cooperate to obstruct TAMs in several different ways. According to the latest research, dextran-coated iron oxide nanoparticles can produce reactive oxygen species (ROS) via the iron oxide-mediated Fenton reaction, which regulates the repolarization of TAMs to M1 macrophages and obstructs the growth of BC [102]. By attracting macrophages to the TAMs and inducing M2 polarization to M1, chemotherapy combined with macrophage-related treatment might improve the anti-cancer impact [118]. As discussed above, targeting TAMs in BC presents a promising avenue for revolutionizing cancer therapy. Strategies aimed at reprogramming TAMs, limiting their infiltration, and combining therapies with immunomodulatory approaches show great potential in enhancing treatment efficacy and improving patient outcomes [102,105].

## 4. Cytokine Expression in the Breast Cancer Microenvironment

The BC microenvironment is a complex network of cells, including cancer cells and stromal and immune cells, all communicating with each other through cytokines. They play critical roles in influencing tumor growth, invasion, metastasis, and the immune response against the tumor [119]. More specifically, cytokines, also known as immunomodulatory agents, are produced in both healthy and unhealthy settings and are released by a variety of cell types, comprising immune cells, immunocompetent cells (such as adipocytes), and certain cancer cells [120]. Based on the microenvironment, they can function as either pro-inflammatory or anti-inflammatory and pro-tumorigenic or anti-tumorigenic effectors and participate in both type 1 and type 2 cellular immunity [26]. Through binding to their surface receptors and subsequent activation of various signaling pathways, cytokines can influence the behavior of other cells [121]. Several cytokines, such as chemokines, interleukins (ILs), adipokines, transforming growth factors (TGFs), tumor necrosis factor (TNF), colony-stimulating factors (CSFs), and interferons (IFNs), can act singly, synergistically, protagonistically, or antagonistically to control the immune and inflammatory responses [120].

Chemokines are chemoattractant cytokines that deliver chemical instructions to attract inflammatory cells, such as leukocytes (neutrophils and monocytes), as well as other cell types, such as endothelial and epithelial cells, to the location of interest [121]. These cytokines can be either homeostatic (movement and location of cell subsets) or inflammatory (e.g., CXCL8 and CCL3), attracting cells through an inflammatory stimulus. BC may be promoted by CCL2, CCL5, CXCL8, and CXCL12, although it may be inhibited by CXCL9, CXCL10, and CCL16 [122]. In addition, ILs are low molecular weight cytokines, which also possess both pro- and anti-inflammatory characteristics [121]. CXCL12 can attract immune cells to the tumor microenvironment, potentially enhancing anti-tumor responses and improving the prognosis [123]. Other specific chemokines, such as CXCL8, can contribute to tumor progression by promoting angiogenesis and metastasis, leading to a poorer prognosis [124].

Moreover, adipocytes, pre-adipocytes, macrophages, stromal cells, fibroblasts, and endothelial cells are all components of adipose tissue, which also secretes adipokines, (adipocytokines) [125]. They can control energy expenditure, inflammation, malnutrition, and fat distribution, but they can also accelerate the development of cancer, metabolic illnesses, and low-grade inflammation linked to obesity [126]. Adipokines can be divided into two categories: pro-inflammatory (IL-10 and adiponectin) and anti-inflammatory (leptin, TNF, IL-1, IL-6, and IL-8), which connect obesity and inflammation [127]. Low levels of adiponectin, which has anti-inflammatory and anti-proliferative properties, are associated with poorer prognosis and an increased risk of BC development [128]. Conversely, elevated leptin, known for its pro-inflammatory and pro-angiogenic effects, has been linked to a higher risk of aggressive BC and a worse prognosis [129].

Another crucial category, TGF, a member of the epidermal growth factor (EGF) family, promotes cell growth, stimulates the development of epithelia, and has a role in cancer and angiogenesis [130]. TGF-β is a multifunctional cytokine that can have both tumor-suppressive and pro-tumorigenic effects depending on the BC stage and TME [131]. In early-stage BC, higher levels of TGF-β have been associated with a favorable prognosis, as it can inhibit cell proliferation and promote differentiation [132]. However, in advanced stages, TGF-β often switches to a pro-tumorigenic role by promoting tumor progression, immune evasion, and metastasis [133]. Therefore, the prognostic significance of TGF-β in BC depends on its context within the disease progression, highlighting the need for a nuanced understanding of its role in accurate prognosis and potential therapeutic interventions [134].

Finally, TNF is a crucial cytokine involved in the production of the pro-inflammatory response and different cellular responses, and CSF has been linked to BC [135]. More specifically, TNF-α plays a crucial role in cell survival, proliferation, tumor-promoting, aggressiveness, macrophage infiltration, CAF phenotype, inflammatory chemokine expression, and angiogenesis in BC [136]. Its pro-metastatic role requires tumor cell migration for metastasis, and over-expression is associated with aggressive behavior and poor prognosis [120]. TNF-α and IL-1β are essential pro-inflammatory cytokines found in tumor metastasis. Poor prognosis and a higher chance of recurrence are linked to inflammation [121]. ΤNF-α production by peripheral blood T cells in inflammatory BC patients is positively correlated with tumor cells expressing EMT markers, as it is recognized by various stromal cells, including TAMs, adipocytes, epithelial, and cancer cells [137].

Cytokines can activate or modify anti-tumor responses and are essential for the development and operation of different cell types [138]. BC cells communicate with the adipose microenvironment, which communicates with endothelial, cancer-associated fibroblasts (CAFs), immunocompetent, and invading immune cells [120]. The TME contributes to the growth, division, and metastasis of malignant cells, which play a crucial role in the formation of tumors. Some cytokines (such as IL-1β, IL-6, IL-8, IL-10, IL-17, IL-23, leptin, and TGF-β) promote BC invasion and proliferation while others suppress it (such as IL-2, IL-12, and IFNs) [139]. These cytokines contribute to the tumor-promoting inflammation that is a cancer hallmark [125]. Highlighting specific instances, IL-1 is a significant upstream cytokine that is essential for cancer therapy because it reduces metastasis, invasiveness, and inflammation-mediated immunosuppression [140].

Recent advancements in cancer research have shed light on the crucial role that CAFs play in orchestrating immune responses within the TME. These stromal cells, often abundant in BC, are recognized as significant contributors to the complex interplay between cancer cells and the immune system [141]. CAFs exert both immunosuppressive and immunomodulatory effects, secreting cytokines, chemokines, and growth factors, which creates a complex network of signaling pathways [142]. Moreover, they can recruit immune cells and promote an anti-tumor immune response. CAFs also have the ability to remodel the ECM, providing structural support and regulating immune cell movement [143]. Understanding CAFs’ interactions is crucial for developing effective immunotherapeutic strategies for BC. Exploring CAFs as a biomarker or therapeutic target is essential for identifying predictive biomarkers and designing precision medicine approaches.

In BC, specific cytokine profiles can provide valuable insights into a patient’s prognosis. Elevated levels of certain cytokines, such as IL-6 and TNF-α, have been associated with poor prognosis, indicating a more aggressive cancer phenotype and increased risk of metastasis [144]. Conversely, a favorable prognosis may be associated with higher levels of anti-tumor cytokines, such as IFN-γ [145]. More efficient treatment outcomes are associated with IL-6 downregulation and tocilizumab; an IL-6R neutralizing antibody has been demonstrated to block IL-6 signaling in BC [146]. A current study by Sparano et al. demonstrated that a considerably increased risk of distant recurrence is linked to higher levels of the cytokine IL-6 at diagnosis in HER2- patients [138]. Aggressive BC is also linked to the presence of high amounts of IL-17A in the BC microenvironment [147,148].

A relationship between the inflammatory response that promotes BC tumor growth and the tumor-infiltrating capacity of adaptive immune surveillance is made possible by IL-23, which serves many functions in carcinogenesis by suppressing anti-tumor effector immunity [120]. Apart from the cytokines mentioned above, IL-12 seems to be an ideal alternative for immunotherapy-based therapies that might slow tumor development by triggering efficient anti-tumor immune responses and cytotoxic NK and CD8+ T cells that target and destroy tumor cells [149]. Depending on the TME and the quantity of IL-10 receptors expressed on immune cells, IL-10 has an impact on how the tumor immune response is modulated. It benefits angiogenesis, tumor development, tumor escape, and metastasis. IL-10 levels induce EMT, boost TGF-a excretion in macrophages and Treg cells, and inhibit T cell proliferation and activity in BC [150,151]. The therapeutic resistance to radiation, chemotherapy, and immunotherapy is largely attributed to IL-10 generated by TAMs and activation of the IL-10/STAT3/BCL-2 signaling pathway [120,150]. These network relationships are not only dictated by treatment but also by environmental factors, such as endocrine-active substances (EASs) and endocrine-disrupting chemicals (EDCs) that, by altering the activity of the immune system, can contribute to setting an unhealthy pro-tumor inflammation (PTI) (directly correlated to TNF-α and monocytes/macrophages action) [38,152,153,154], thus facilitating the oncogenic process via the promotion of tumor growth, survival, and metastasis and suppressing the anti-tumoral immune response, shaping pivotal TME characteristics [39].

## 5. Immune Evasion in Breast Cancer

As highlighted previously, TILs play a pivotal role in immune evasion mechanisms. They are a diverse population of immune cells that infiltrate the TME, consisting mainly of T cells, B cells, and NK cells [40]. Their detailed role involves recognizing and attacking cancer cells, thereby exerting anti-tumor effects. However, despite their presence, BC cells can employ various strategies to evade immune surveillance and promote tumor growth [155]. One such mechanism is the upregulation of immune checkpoint molecules, such as PD-L1, which interacts with its receptor PD-1 on TILs, leading to T cell exhaustion and impaired anti-tumor responses [56]. Additionally, BC cells can secrete immunosuppressive factors and develop an immunosuppressive microenvironment that inhibits TILs’ function. These processes collectively contribute to immune evasion and tumor progression in BC (Figure 1) [156].

Typically, BCs of any genetic subtype that include more than 50–60% TILs in the tumor or stroma indicate a favorable prognosis [157]. Nevertheless, the nature of the tumor infiltration may play opposing and seemingly paradoxical functions in generating a milieu that is either tumor-antagonizing or tumor-promoting [156]. This is another characteristic of BC that may contribute to the perception that it is an immunologically “silent” tumor, while new research has started to illuminate the relevance of TILs and may eventually show immune cell-specific significance [158]. Moreover, the presence of TILs in the post-neoadjuvant scenario of residual TNBC (which displays high rates of metastatic recurrence) was recently shown to be strongly correlated with the Ras/MAPK signaling pathway, PD-L1 [159]. They discovered that increased cell-cycle pathway activity and, to a lesser extent, increased Ras-MAPK activation both predict a decreased TIL phenotype in the remaining cancer [156].

Furthermore, TAMs are a type of immune cell that infiltrates the TME and can be polarized into different subtypes, such as M1 and M2 [87]. Their detailed role involves complex interactions with cancer cells and the TME, impacting tumor progression and therapeutic responses. However, in the context of immune evasion, TAMs can adopt an M2-like phenotype, promoting tumor growth and metastasis while suppressing the anti-tumor immune response [136]. These M2-like TAMs secrete immunosuppressive factors, such as TGF-β and IL-10, which dampen T cell activity and promote immune tolerance [156]. Moreover, TAMs can facilitate the recruitment of Tregs that further suppress the anti-tumor immune response. Additionally, TAMs can promote angiogenesis and tissue remodeling, developing an immunosuppressive microenvironment that shields cancer cells from immune attacks [136].

Immune evasion, a crucial component of immunoediting, has recently come to be recognized as a potential characteristic of BC. In somatic cells, gene alterations sometimes happen spontaneously or because of the combined action of several carcinogenic stimuli. While some altered cells may self-repair, others go through apoptosis [160]. Neoantigens are expressed on the membrane surface of cells with specific mutations [156]. Before tumors form, these altered cells with neoantigens are recognized as heterologous components, which are eliminated by innate and adaptive immune system components [161]. Tumor-associated antigens (TAAs) can be divided into three categories: autoantigens or embryonic antigens, which are overexpressed or abnormally expressed, modified autoantigens, which are only expressed in the presence of tumors, and neoantigens, which are produced as a result of gene mutations, chromosomal abnormalities, and viral transformation [155,156].

BC cells employ several strategies to escape immune recognition and attack [136]. Specifically, BC can downregulate or modify the expression of major histocompatibility complexes (MHCs) molecules, which are essential for presenting antigens to T cells [158]. This hinders the ability of the immune system to identify cancer cells as abnormal and mount a targeted immune response against them. Some BC cells may completely lose MHC class I expression, rendering them invisible to cytotoxic T cells, which normally recognize and destroy cells presenting foreign or abnormal antigens [162]. The loss of MHC-I and MHC-I APM in BC may reduce immunological responses because MHC-I molecules are important for T cells to present and recognize antigens [161]. When BC cells downregulate or lose MHC expression, they can evade T cell recognition and subsequent destruction [160,162].

**Figure 2:**
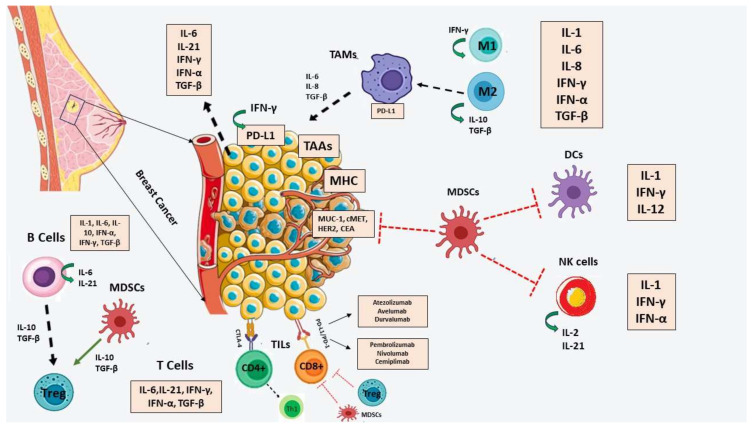
“Unraveling Immune Evasion in Breast Cancer: TAMs, TILs, and Beyond”. Within the complex microenvironment of BC, this intricate graphic sheds light on the strategies cancer cells employ to evade the vigilant immune system. TAMs, depicted in a spectrum from M1-like pro-inflammatory to M2-like immunosuppressive states, interact intimately with cancer cells. Their secreted factors develop an immunosuppressive shield, restraining the efforts of TILs, which are portrayed as an assembly of CD8+ cytotoxic T cells and CD4+ helper T cells, to recognize and eliminate cancer cells. Highlighted in the graphic is the engagement between PD-1 receptors on TILs and PD-L1 ligands on cancer cells and TAMs. This interaction, while a natural regulatory mechanism, can be exploited by cancer cells to incapacitate immune cells. Monoclonal antibodies targeting PD-1/PD-L1 interactions are depicted, reinvigorating the immune response and rekindling the assault on cancer cells. The challenges posed by immune evasion in the context of metastasis become evident, illustrating the multifaceted nature of the battle against BC. This figure highlights the need for a comprehensive understanding of immune evasion mechanisms and underscores the potential of innovative strategies to outsmart the cancer’s defenses and bolster the body’s immune armory against BC’s relentless advance. Figure key: green arrows denote activation, red dotted line denotes inhibition, and boxes denote cytokines secreted by immune cells or tumors. BC: breast cancer; TILs: tumor-infiltrating lymphocytes; TAMs: tumor-associated macrophages; PD-1: programmed cell death protein 1; PD-L1: programmed death-ligand 1; MDSCs: myeloid-derived suppressor cells; TAAs: tumor-associated antigens; IFNs: interferons; TGF-β: transforming growth factor-beta; IL-: interleukin-; NK: natural killer; Tregs: CD4+ regulatory T cells; HER2: human epidermal growth factor receptor 2; MUC1: mucin 1; c-MET: mesenchymal-epithelial transition factor; CEA: carcinoembryonic antigen; MHCs: major histocompatibility complexes; DCs: dendritic cells.

MHC-I, adhesion, and costimulatory molecules, loss of antigens, and increased expression of immunosuppressive components, such as HLA-G, HLA-E, and PD-L1, as well as other immunosuppressive factors, for instance, cytokines and metabolites that help the body avoid immune recognition, are some of the underlying mechanisms [158]. TAMs, CD8, cytotoxic T cells (CTLs), CD4 lymphocytes, NK, Treg cells, and MDSCs are indications of inflammatory immune cells [155]. Among these, in a study by Vasaturo et al., Treg cells, MDSCs, and macrophages play a major role in the immunosuppressive activity by releasing important chemicals including TGF-β, prostaglandin E2, indoleamine 2,3-dioxygenase (IDO), and IL-10 [163]. For instance, TGF-β inhibits T cell activation and encourages Treg development, resulting in an environment that is immunosuppressive and favorable for tumor growth [164]. Dendritic cells’ capacity to deliver antigens and activate T cells can be decreased by IL-10’s ability to prevent their development. On the other hand, IDO depletes tryptophan, causing T cells to become apoptotic and lose their ability to function [136]. In contrast to CTLs, which are linked to a good prognosis, The abundance of Tregs, MDSCs, and TAMs in the stroma also aids cancer cells in evading immune surveillance and is associated with a poorer prognosis [22,160]. Increased PD-L1 expression and the nuclear factor (NF)-kb have a role in immune evasion and TNBC development [165]. In conclusion, BC cells employ a range of mechanisms to evade immune surveillance and destruction, allowing them to escape immune detection and elimination (Figure 2) [156].

## 6. The Role of Immune Checkpoints in Breast Cancer

Immune cell surface receptors, commonly referred to as immune checkpoints, have a crucial function in regulating the process of the immune response’s activation or inhibition. They additionally perform an essential part in maintaining tolerance for themselves and limiting excessive immune activation [29]. The initial therapeutic efficacy of checkpoint inhibition was demonstrated in melanoma, and approvals for its use in other T cell inflammatory malignancies followed shortly. However, previously, it was thought that BCs were immunogenically inactive and that immune treatment was ineffective against them [31]. To boost the immune response against malignancies, immune CIs, a type of immunotherapy, block the cell surface receptors that are located on T cells [31]. Tumor cells and tumor-infiltrating immune cells can express these molecules, contributing to the establishment of an immunosuppressive TME that hampers effective anti-tumor immune responses [166]. In BC, the expression and significance of CIs have emerged as key areas of investigation to identify novel therapeutic targets and improve patient outcomes [167]. Several CIs have been extensively studied in BC, mainly including programmed cell death protein 1 (PD-1), programmed death-ligand 1 (PD-L1), cytotoxic T-lymphocyte-associated antigen 4 (CTLA-4), and lymphocyte-activation gene 3 (LAG-3) [168]. Inhibitory receptors like T cell immunoglobulin and mucin 3 (TIM-3) and V-domain Ig suppressor of T cell activation (VISTA), activating molecules like OX40 (CD134) and glucocorticoid-induced TNFR-related protein (GITR), and other targets have all been explored [167,169].

Examining each checkpoint individually, CTLA-4, a member of the immunoglobulin superfamily, interacts with ligands B7-1 and B7-2 to negatively inhibit T cell activation [167]. The CTLA-4 checkpoint function is necessary for the immune system to prevent both autoimmune reactions and uncontrolled immunological responses. The interactions between CTLA-4 and B7 prevent T cells from activating early in secondary lymphoid organs [170]. Similar to CD28, CTLA-4 binds more strongly and scavenges CD28 ligands, which results in unfavorable signaling. Because CTLA-4 functions in the early stage of the T cell response in lymph nodes during tumor immunoregulation, it is possible for uncontrolled proliferation to occur in the absence of CTLA-4 [167,171]. The first therapy to be licensed for melanoma was ipilimumab, a human IgG1 monoclonal antibody against CTLA-4, in 2010. It significantly increased survival, had a sustained response, and may have a long-term disease control [172]. Later research has shown that the CTLA-4 signaling pathways in BC were more elevated in invasive ductal carcinomas than in ductal carcinoma in situ [173]. It backs up the idea that an immune system is suppressive during the evolution of an invasion. Finally, a recent in vitro study has demonstrated that exposure to a TNBC cell culture causes mononuclear blood cells to produce more CTLA-4 [170,174].

In addition, the immunoglobulin superfamily member PD-1 is essential for the signaling of programmed cell death in response to T cell-mediated events. In the TME, it is more broadly expressed than CTLA-4 and may be found in a variety of immune cell types [175]. T cell inhibition and fatigue occur because of the inhibitory signal sent by PD-1 when it binds to its ligand, PD-L1. At later phases of tumor progression, PD-1 mostly limits T cell activation in peripheral tissues [176]. Following the FDA’s approval of pembrolizumab, nivolumab, and cemiplimab as PD-1 inhibitors due to the successful antitumor T-cell responses to PD-1 inhibition in several cancer models [177], pembrolizumab, a humanized IgG4 mAb, was initially approved for metastatic melanoma and non-small-cell lung cancer. It has since received approvals for various tumor types, including squamous cell carcinoma, solid tumors with high microsatellite instability, advanced gastric cancer, cervical cancer, urothelial carcinoma, TNBC, and tumors with high mutational burden [167]. In some cases, pembrolizumab has demonstrated durable responses and improved overall survival rates in advanced or metastatic BC patients who have exhausted other treatment options [178,179]. Last but not least, PD-1 inhibits activated immune cells by interacting with PD-L1 and PD-L2 ligands, which are extensively expressed in a variety of immune cells and tumor types [178]. The PD-1/PD-L1 pathway is used by tumors to suppress T cell-mediated immunity, which promotes aberrant cancer cell growth. Because of this relationship, PD-L1 is a desirable target for immunotherapy. Atezolizumab, durvalumab, and avelumab are the three PD-L1 inhibitors that the FDA has authorized [166].

The expression patterns of CIs in BC have significant prognostic implications. More specifically, high PD-L1 expression on tumor cells and tumor-infiltrating immune cells has been associated with more aggressive tumor phenotypes, increased risk of recurrence, and reduced overall survival [180]. Particularly, PD-L1 over-expression is found in 9–45% of HR+ early BC patients, with a decrease in the metastatic stage. In nonmetastatic HER2+ BC, the positivity rate is 30–35% and 35–60% in early TNBC, compared to 9–15% in advanced BC and 30–40% in metastatic TNBC [171]. A recent systematic review and meta-analysis showed increased positivity rates in primary tumors compared to metastasis in immune cells and tumor cells/immune cells, compared to tumor cells [179]. Moreover, elevated expression of other checkpoint molecules, such as LAG-3, has been linked to poor clinical outcomes. These findings underscore the importance of CIs as potential biomarkers to predict disease progression and therapeutic responses [180].

CIs, such as PD-1/PD-L1 and CTLA-4 inhibitors, have shown remarkable clinical efficacy in subsets of BC patients, particularly in those with TNBC and HER2+ BC [177]. CTLA-4 inhibitors can stop T cell depletion and increase the anti-tumor T cell response by preventing the interaction between CTLA-4 and CD80/86 ligands [167,176,179]. PD-1 interrupts effector T cell responses by interacting with its ligands PD-L1 and PD-L2 [175]. According to other research, inhibiting CTLA-4 in mice can prevent the growth of tumors and develop immunological memory [181]. This prompted clinical studies and the advancement of humanized monoclonal antibodies to block CTLA-4 interaction in cancer patients [167]. As a result of an intriguing study by Honjo et al. that demonstrated that PD-1/PD-L1 binding causes T cell fatigue, several humanized antibodies have been discovered, and clinical trials in advanced cancer patients have been conducted [182].

BC has been successfully treated using CIs, although only 20–40% of patients see tangible advantages. Clinical trials exploring the efficacy and safety of these novel agents have yielded promising results in the management of BC [167,183]. CIs, such as pembrolizumab and atezolizumab, work by blocking key proteins that dampen the immune response, enabling immune cells to recognize and attack cancer cells more effectively [167]. In combination with nab-paclitaxel chemotherapy, atezolizumab showed significant improvement in progression-free survival in patients with advanced TNBC, leading to its approval as a treatment option for this specific subtype [179,184]. To choose patients who are responsive while minimizing costs and adverse effects, researchers need to understand treatment response variability and discover predictive biomarkers [171].

## 7. Other Immunotherapy Approaches for Breast Cancer Patients

The survival rate and quality of life of BC patients have recently increased thanks to surgery, radiation, chemotherapy, endocrine treatment, targeted therapy, and immunotherapy [185]. However, the established resistance in patient therapy restricts therapeutic efficacy and treatment outcomes, contributing significantly to the high mortality rate in BC [186]. Therefore, there is a critical need for novel therapeutic approaches to combat immune evasion, enhance patient quality of life, and improve BC survival [187]. Immunotherapy harnesses the body’s immune system to recognize and target cancer cells, offering the potential for targeted and durable treatment outcomes. It includes varied tactics, such as adoptive cell treatments (ACTs), vaccinations, oncolytic viruses, and, most significantly, immune checkpoint inhibition, which has attracted researchers’ interest since it has the potential to be one of several revolutionary therapeutic options [188]. To comprehensively explore the intricate relationship between BC immunotherapy and TME, it is imperative to consider some relevant studies that shed light on this critical aspect of cancer research. Several noteworthy studies have contributed to our understanding of this complex interplay and offer valuable insights into various facets of BC immunotherapy and its influence on the TME (Clinical Trials Table 3). These studies have explored diverse aspects ranging from novel therapeutic approaches and CIs to the role of specific immune cell populations within the TME [189,190,191,192,193].

### 7.1. CAR-T Cell Therapy

CAR-T cell therapy involves identifying and isolating T lymphocytes from peripheral blood or tumor sites. These cells are then modified, activated, and expanded ex vivo before being reintroduced into the patient [204]. CAR-T falls into three categories: TIL-based treatments, T cell receptor (TCR) gene therapy, and CAR-T. Patients with advanced hematological malignancies have previously experienced protracted responses and remissions because of the latter technology [205]. By recognizing antigens in self-tumor cells, CAR-T cells are equipped with antibodies that have harmful effects. The therapeutic effects of CAR-T cell treatment, including BC, have not lived up to expectations in solid tumors despite recent triumphs in treating hematologic malignancies [187].

Peripheral blood mononuclear cells (PBMCs) are the first step in the development of CAR-T cells, either from patients for autologous CAR-T therapy or from healthy third parties for allogeneic CAR-T therapy [206]. Since CAR-Ts lack MHCs, they can detect cancer cells by targeting cell membrane-expressed antigens through antibody-derived targeting domains connected to T cell activation domains [207]. Additionally, CAR-Ts are thought to be the best TIL substitute since they are simple to scale up to clinically useful sizes [208]. Effective CAR-T cell targets for the therapy of BC patients have been discovered, including HER2, MUC1, and mesothelin [209]. ACTs have new prospects due to the discovery of neoantigens and the use of other immune cell types like NK cells or DCs [205]. Moreover, utilizing viral or non-viral vectors (like retroviruses), CAR is delivered into T cells and integrates genes utilizing techniques like transient mRNA transfection and synthetic DNA transposon systems [207].

In BC, CAR-T therapy is being investigated for targeting antigens, such as HER2 and ERBB2 [185]. Clinical trials evaluating CAR-T targeting HER2 have shown promising preliminary results in patients with HER2+ BC. TNBC has several antigens that CAR-T specifically targets, for example, more than 90% of human TNBCs have isoforms of the tumor-associated MUC1 antigen (known as tMUC1) [210]. A second antigen, mesothelin, is expressed by around 70% of primary TNBC but is undetectable in non-cancerous breast epithelium [211]. Additionally, additional antigens such as brachyury and the folate receptor alpha (FRα) have been researched for CAR-T cells in TNBC [212]. Combining ACT with targeted treatments could be considered a valuable approach for future therapeutic efforts, particularly in the case of TNBC, despite the associated challenges [185].

Tumor heterogeneity presents challenges for CAR-T cell therapy due to the variability in antigen expression on tumor cell surfaces [208]. Multitarget CAR-T cells have shown improved anti-tumor activities in preclinical BC studies, but selecting multiple targets remains a challenge. Toxicity is a significant challenge in CAR-T cell therapy, with the following six types: on-target, on-target/off-tumor, off-target, neurotoxicity, genotoxicity, and immunogenicity [204]. To address toxicity, various approaches have been developed, including multitarget CARs, affinity-tuned CARs, inhibitory CARs (iCARs), suicide genes, and transient RNA-expressing CARs [185]. These approaches improve safety and differentiate tumors from normal cells, but their effects depend on target expression levels and may interrupt T cell anti-tumor effects [187,207]. Suicide genes, like HSV-TK, iCasp9, and CD20, can control cytotoxicity but can also have weaknesses, such as unintended elimination of modified functional CAR-T cells, immunogenicity, and a long time to thake effect [213].

### 7.2. Therapeutic Cancer Vaccines

Oncolytic viruses (OVs) and CVs constitute a cutting-edge and promising treatment strategy. The foundation of CVs treatment is the immune system’s capacity to distinguish between antigens produced improperly in tumor cells and those found on the surface of healthy cells [34,214]. To induce immune responses against TAAs, several methods, including bacterial, viral, peptide, or gene-based CVs, are being investigated in clinical studies. Even though no overall effect was seen in pertinent phase 3 clinical trials, several investigations studying therapeutic CVs in BC are still in progress [213]. Regarding Ovs, this approach is predicated on inducing systemic anti-tumor immunity while selectively infecting and killing tumor cells with little to no impact on healthy tissue [213,215]. By boosting immune cell infiltration and promoting subsequent cascade immunological networks, Ovs can be genetically modified to express immune cytokines and enhance immune responses. Therapy with Ovs is now a growing area of research [215].

Therapeutic CVs are designed to stimulate the patient’s immune system to recognize and attack cancer cells. These vaccines typically contain specific antigens or antigen fragments that are present in cancer cells [216]. When administered, the vaccine primes the immune system to recognize these antigens as foreign and initiate an immune response against cancer cells expressing them [205]. Depending on the type of antigen, there are many CV types. The most investigated are those made with TAA peptides, while additional antigens being researched to promote both innate and adaptive anti-tumor immunity include those made with tumor protein- or carbohydrate-associated antigens, as well as those based on DNA and DCs [217]. Moreover, CVs have only had sporadic clinical success throughout testing. All phase 3 clinical studies so far have been negative for therapeutic BC vaccines, which were initially examined in metastatic disease and later in the adjuvant context [218]. As disease loads and immune suppression are both low, there is growing interest in using CVs for disease interception and prevention [219].

Mentioning some specific examples, a MUC1-targeting vaccination was found to provide protection from recurrences in a different clinical study for patients with ER+ stage II Bca (12.5% vs. 60% recurrences in the vaccine vs. placebo groups, respectively) [220]. Clinical experiments have utilized immunogenic peptides generated from HER2 that contain sequences from various extracellular, intracellular, and transmembrane domains and have demonstrated immunological and clinical responses [187,221]. Furthermore, an E75 immunization significantly decreased the disease recurrence rate and disease-free survival (DFS), but had no meaningful impact on overall survival at 59 months (OS59), according to a recent meta-analysis of 24 clinical trials including a total of 1704 vaccine recipients and 1248 control participants [214]. However, the perplexing narrative of HER2 vaccination continues, leaving one to wonder how a vaccine targeting HER2 could potentially be beneficial in treating individuals with HER2- tumors but not HER2+ tumors [205]. Finally, therapeutic CVs have been developed to target TAAs, such as mammaglobin-A and HER2. While the efficacy of therapeutic vaccines in BC is still being studied, early-phase clinical trials have shown encouraging results, with some patients experiencing immune responses and disease stabilization [222].

### 7.3. Immune Modulators

Researchers are exploring new biomarkers to target in combination with immunotherapy, such as IDO, OX-40 (TNF receptor member), and glucocorticoid-induced TNFR-related protein (GITR) [223]. Interestingly, GITR has been proposed as a possible therapeutic target for immunotherapy with important key features regarding Tregs in the tumor context also [224,225,226,227]. IDO, an immunosuppressive molecule, has been linked to anti-tumor responses when used alongside anti-PD-1 therapy [228]. Apart from CIs, other immune modulators are being explored to enhance the anti-cancer immune response. As described previously, cytokines, such as ILs and IFNs, are signaling molecules that play a crucial role in immune regulation [122]. These immune modulators can be administered to stimulate the immune system and promote an anti-tumor immune response. Additionally, small molecule inhibitors that target immune regulatory pathways are under investigation. Combining these immune modulators with other therapies, such as chemotherapy or targeted therapies, may enhance their effectiveness in BC treatment [229].

### 7.4. Combination of Therapies

Given the complex nature of BC, combination therapies have garnered significant attention. These combinations often involve immunotherapies paired with traditional treatments like chemotherapy or radiation. Chemotherapy, for example, can induce immunogenic cell death, promoting an immune response. Radiation therapy can develop an immune-stimulatory microenvironment, enhancing the effectiveness of immunotherapy [230]. Combination chemotherapy may have benefits including improved efficacy and the ability to reduce doses while maintaining or increasing efficacy, less toxicity, and a slowed or delayed emergence of drug resistance [231].

Furthermore, OX40 (CD134) is a co-stimulatory protein that supports the inhibition of Tregs and the expansion of immune cells. In 30% of mouse cancer models, tumors were reduced by PD-1 blockers and OX40 agonists [232]. A phase II clinical trial for TNBC patients has assessed anti-OX40 in conjunction with an anti-PD-L1 antibody [3]. Many people who additionally use turmeric have been investigated utilizing CTLA-4 inhibitors and CAR-T cells [229]. Additionally, 71% of TNBC and ER+ patients in a phase II therapeutic study who simultaneously suppressed CTLA-4 and PD-1 benefited [233]. Moreover, trastuzumab, which promotes ADCC, is the most popular immunotherapy strategy in British Columbia, according to several studies [210]. In a phase III clinical study, individuals with HER2+ BC received either trastuzumab or the HER2 vaccination. Positive outcomes for BC patients were seen when anti-4-1BB agonist and anti-PD-L1 mAb were combined [217]. In the animal model of BC, trastuzumab with an anti-4-1BB agonist antibody increased the NK cytotoxicity. Combining immunotherapies with targeted therapies, such as HER2-targeted agents, can lead to more comprehensive and durable responses in HER2+ BC patients [231].

### 7.5. Novel Therapies

The KEYNOTE-522 trial represents a groundbreaking advancement in the treatment of TNBC, reshaping the landscape of clinical practice [234]. In current literature, few pivotal studies investigated the efficacy of combining immunotherapy with chemotherapy, specifically, the anti-PD-1 checkpoint inhibitor pembrolizumab, in the neoadjuvant setting for TNBC patients [234,235,236]. The results were nothing short of remarkable, demonstrating that this combination therapy substantially improved pCR rates, which is a strong predictor of long-term outcomes. In crucial trials, 64.8% of patients who received pembrolizumab in addition to chemotherapy achieved pCR compared to 51.2% of those treated with chemotherapy alone. This significant increase in pCR rates has ushered in a new era of hope for TNBC patients, suggesting that immunotherapy holds immense promise in transforming the management of this aggressive subtype of BC [235,237].

In more recent years, the therapy of BC has been altered by antibody-drug conjugates (ADCs). ADCs combine the specificity of monoclonal antibodies with the cytotoxic power of chemotherapy, delivering potent anti-cancer agents directly to tumor cells while sparing healthy tissue [238]. One notable ADC is sacituzumab govitecan, which has shown remarkable results in clinical trials [239,240]. In the pivotal ASCENT trial, sacituzumab govitecan demonstrated impressive efficacy in patients with metastatic TNBC who had previously received multiple lines of therapy. The trial reported a median progression-free survival (PFS) of 5.6 months and an OS of 12.1 months for patients receiving sacituzumab govitecan, compared to 1.7 months and 6.7 months, respectively, for those in the control group [239,241]. These findings underscore the significant impact of ADCs in reshaping the treatment landscape for BC, particularly for those with challenging-to-treat subtypes, such as TNBC.

Moreover, PARP inhibition has emerged as a promising strategy in BC treatment, particularly for patients with BRCA mutations. Clinical trials have shown significant progress in harnessing PARP inhibitors, such as olaparib and talazoparib [242,243]. The OlympiAD trial, for instance, demonstrated that olaparib significantly extended PFS in BRCA-mutated metastatic BC patients compared to chemotherapy. The trial reported a median PFS of 7.0 months with olaparib versus 4.2 months with chemotherapy [244]. Similarly, the EMBRACE trial revealed that talazoparib conferred a substantial improvement in PFS compared to chemotherapy for BRCA-mutated metastatic BC patients, with a median PFS of 8.6 months with talazoparib versus 5.6 months with chemotherapy [245,246].

Since it has been linked to a worse clinical stage, a heavier burden from metastatic disease, and a worse response to immunotherapies, MDSC growth is one of the main immunosuppressive strategies utilized by BCs [231]. Different immunotherapeutic approaches are currently being used in BC to target MDSCs, either by lowering their numbers or blocking their immunosuppressive effects [247]. One of the methods being investigated in BC to decrease the MDSC niche is blocking the generation of cytokines and chemokines that encourage the growth and migration of MDSCs to the TME [217].

BsAbs are another sort of synthetic molecule that is produced to detect two distinct epitopes or antigens on immune cells and tumor cells, enabling the immune system to target these cancer cells [248]. There are several BsAbs pertinent to BC in development 84. When used in conjunction with chemotherapy, the BsAb zanidatamab, which targets two distinct HER2 epitopes, was well-tolerated and had anti-tumor effectiveness in BC patients with metastatic HER2-amplified disease [249]. BsAbs from a broad range of tissue-independent targets, including CD3, CEACAM5, epithelial cell adhesion molecule (EpCAM), EGFR, mesothelin, and Trop2, are being researched in TNBC [205,248].

Finally, immunotherapy’s clinical results are highly variable and contentious among cancer patients. These variances are brought about by variations in the TME, cytokine balance, and genetic makeup of the malignancies, as well as the immunological state of the patients [187,213,223]. The immunological health of the patients affects the effectiveness of cancer immunotherapy. Diversities in HLA among people result in a variety of reactions. As a result, HLA polymorphisms must be considered as important variables in patient responses to immunotherapy [217]. TNBC has increased genetic instability and a larger likelihood of discovering neoAgs than other BC subtypes, which number in the dozens. For TNBC patients, many tailored vaccination strategies (such as DNA, RNA, and peptide vaccines) have been examined in clinical studies [250]. As was already mentioned, tumors are heterogeneous and have a variety of molecular patterns, and each form of cancer requires a different sort of therapeutic strategy [205]. NeoAg-peptide vaccinations alone are inadequate for eliciting immunological responses since only 5–20% of neoAgs can produce CTLsTA. Thus, they should be used in conjunction with other immunotherapy techniques [251].

## 8. Biomarkers and Future Directions

**Figure 3:**
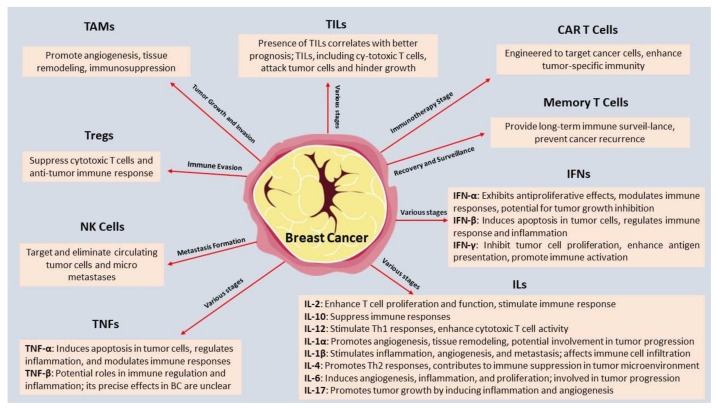
“Immune Cell Interactions in Different Stages of Breast Cancer”. This figure illustrates the diverse roles of various immune cells, including TILs, TAMs, Tregs, NK cells, CAR T cells, and memory T cells, and the impact of different types of IFNs, ILs, and TNFs in different stages of BC (arrows). Please note that the interactions are complex and multifaceted and are influenced by the intricate interplay of immune components within the tumor microenvironment. Various stages means at least two 2 stages of the following: Stage 0 (early stage; carcinoma in situ), Stage I-II (Stage I: the tumor is smaller and has not spread to the lymph nodes; Stage II: the tumor may be larger or have spread to a few nearby lymph nodes), Stage III (the tumor is larger and may have invaded nearby tissues), and Stage IV (metastatic breast cancer). BC: breast cancer; TILs: tumor-infiltrating lymphocytes; TAMs: tumor-associated macrophages; Tregs: regulatory T cells; NK: natural killer cells; CAR: chimeric antigen receptor T cells; IFNs: interferons; ILs: interleukins; TNF: tumor necrosis factor.

In recent years, biomarker-based immunotherapy has emerged as a promising approach for BC management. Immunotherapies, such as immune CIs, have demonstrated remarkable success in several cancer types, but their application in BC has been more complex [252]. Each subtype exhibits distinct immune profiles and treatment responses, complicating the identification of universal biomarkers for immunotherapy [253]. As assumed in previous sections, the BC microenvironment comprises various immune cell populations, stromal cells, and cytokines that influence treatment response. All of these molecules can affect different stages of BC, as detailed in Figure 3. TME complexity hampers the reliable identification of predictive biomarkers and may lead to immune resistance [205]. Consequently, clinical trials investigating biomarker-based immunotherapies in BC have faced challenges in trial design and endpoints. Variability in patient selection criteria, treatment regimens, and response assessment methods can impact trial outcomes and the comparison of results across studies [52,254].

Although PD-L1 expression has been extensively studied, its predictive value remains inconsistent. Future research should focus on identifying novel biomarkers and validating their clinical significance. Since PD-L1 expression is highly heterogeneous, it can change rapidly on either immune or malignant cells [165]. In neoadjuvant first-line therapies, the combination of immune CIs plus chemotherapy/targeted medicines has previously shown clinical benefit and promise [34]. For metastatic TNBC in the first and second lines of therapy, high counts of TILs and CD8 T cells indicate a favorable response to immune CIs. It is essential to initially evaluate CD8 T lymphocytes in ER- tumors [3]. Therapy with immune CIs should be considered when immune CIs are present. According to evidence, the combination of durvalumab and tremelimumab can boost overall response rates by 23% [255]. The main problem is that there is currently a lack of predictability and reliability in the biomarkers used to identify patients with TNBC, including PD-L1 expression, TILs, and TMB. Therefore, it is urgently necessary to discover new predictive biomarkers employing single-cell, deep multi-omics analysis [35]. So far, the TNBC predictive biomarker study using multi-omics technologies has shown encouraging findings [251].

It is of utmost importance to investigate specific components of the TME, such as unique immune cell subsets, stromal cells, or cytokines. These investigations aim to identify targets that can be leveraged to enhance the response to immunotherapies. Additionally, researchers are exploring combinations of immunotherapies with other treatment modalities, including chemotherapy, radiation therapy, and targeted therapies [256]. This approach is designed to create synergistic effects that can significantly improve patient outcomes. Furthermore, tailoring immunotherapeutic strategies for BC management involves developing patient-specific approaches [25]. This is achieved by taking into account individual immune profiles, genomic alterations, and tumor characteristics [257]. Moreover, investigating the mechanisms behind resistance that may develop in response to immunotherapy is essential. Researchers are actively devising strategies to circumvent or mitigate these resistance mechanisms, making immunotherapy more effective over the long term.

Additionally, combining techniques from immunotherapy, chemotherapy, and radiation holds promise for TNBC patients who have limited long-term treatment options that are both efficient and safe. Even when combined with immune CIs, the abscopal impact observed in preclinical trials has not been successfully translated into clinical reality, raising the question of whether alternative strategies should be considered [158,253]. Moreover, AI is becoming more widely acknowledged as a promising technique for identifying biomarkers, which has improved clinical care [185]. Nevertheless, the development of reliable and consistent computational, clinical, and laboratory techniques must be accomplished concurrently and verified across several cooperating locations to guarantee its effective deployment [52]. High-resolution images of the tumor immune microenvironment are also possible by using cutting-edge technologies, such as single-cell RNA sequencing and CyTOF-based immune profiling. A key component of effective tailored cancer therapy is improving medication response by altering the dynamic tumor ecology [258].

Studies on immunotherapies for long-term immune memory and disease recurrence are crucial for developing durable treatment strategies [254]. Future research should focus on patient-specific approaches based on individual immune profiles, genomic alterations, and tumor characteristics [259]. Combination therapies targeting cancer cells and the immune system offer promising therapeutic interventions [260]. Further research is needed to identify reliable biomarkers, understand resistance mechanisms, optimize treatment sequencing, explore long-term immune memory, and develop personalized immunotherapeutic strategies.

## 9. Conclusions

The immune microenvironment has emerged as a pivotal player in BC progression and response to treatment [213]. This review has highlighted key findings that underscore the significance of understanding the interplay between the immune system and the BC microenvironment. By exploring the impact of immune cells, immune CIs, and cytokines, as well as the potential of combination therapies, this investigation provides valuable insights into the therapeutic implications of targeting the immune microenvironment in BC. As BC is a heterogeneous disease with diverse molecular subtypes, a one-size-fits-all approach to therapy may not be optimal. Tailoring treatment based on individual immune profiles, tumor characteristics, and genomic alterations can lead to more effective and precise interventions [261]. Therefore, a possible candidate strategy for improving the survival of BC, particularly TNBC patients, may involve reducing immunosuppressive chemicals in the TME while concurrently targeting new molecules, especially those implicated in angiogenesis and immune responses.

Traditional treatments, such as chemotherapy and radiotherapy, can modulate the immune microenvironment and enhance the efficacy of immunotherapies [229]. Although immunotherapy is a promising strategy for treating cancer, it can be difficult to manage immune responses because of the many cellular and molecular connections within the immune system. Combination immunotherapy is considered a potential approach for BC immunotherapy since concentrating on only one aspect of the immune system is insufficient [205]. Furthermore, the simultaneous targeting of multiple immune CIs and the incorporation of OVs and CVs hold the potential to overcome resistance and improve treatment responses [207]. Finding new predictive biomarkers of response to immune CIs and combination therapies is crucial to help with patient classification and therapeutic decision-making. Numerous attempts have been made to pinpoint an immunological profile unique to BC patients who respond well to immunotherapy, but conclusive information is still missing. The development of “add-on designs”, which combine a novel immune medicine with a clinically tested modality without producing adequate plans for each individual patient, will be avoided with the use of in-depth translational research and the use of biomarkers [252].

In conclusion, understanding the complex interactions between immune cells, immune CIs, and cytokines provides valuable insights into the therapeutic implications of targeting the immune microenvironment [14,260]. By embracing personalized treatment approaches based on individual immune profiles, we can unlock the full potential of immunotherapies and enhance the prospects of successful outcomes for BC patients. Moving forward, continued research and collaboration will be vital to unraveling the complexities of the immune microenvironment and translating this knowledge into transformative therapies for better BC management.

## Figures and Tables

**Table 1:** Key concepts and findings of the review.

Section	Main Findings/Concepts
Introduction	Introduction to the importance of understanding the immune microenvironment in breast cancer.
Decoding the multifaceted roles of tumor-infiltrating lymphocytes (TILs) in breast cancer	TILs subgroups—distribution, density, and functional characteristics within the tumor—investigating the prognostic value of TILs in breast cancer.
The multifaceted roles of tumor-associated macrophages (TAMs) in Breast Cancer	The presence of TAMs in the breast cancer microenvironment—TAMs subtypes and their effect on tumor progression, invasion, and metastasis—therapeutic strategies targeting TAMs in breast cancer
Cytokine expression in breast cancer microenvironment	Discussion on the role of cytokines in the breast cancer microenvironment.
Immune evasion in breast cancer	Exploration of mechanisms used by breast cancer to evade the immune system.
The role of immune checkpoints in breast cancer	Discussion on the significance of immune checkpoints in breast cancer.
Other immunotherapy approaches for breast cancer patients	CAR-T cell therapy—therapeutic cancer vaccines—immune modulators—combination of therapies—novel therapies
Biomarkers and future directions	Exploration of potential biomarkers and future research directions in breast cancer immunotherapy.

**Table 2:** “Cell Populations in the Tumor Microenvironment (TME)”. This table provides an overview of various cell populations within the TME, highlighting their functions and potential therapeutic targeting. Understanding the roles of these diverse cell types is crucial for developing effective treatments and interventions in the field of oncology. This comprehensive table aids researchers and clinicians in navigating the complex interplay of cells within the TME, offering insights into the potential for targeted therapies and immunomodulation strategies.

Cell Population	Function	Therapeutic Targeting
**TILs**	Immune response against tumors	Immunotherapies
**Stromal TILs (sTILs)**	Organizing TME, cytokine production	Immune modulators
**CD8+ T Cells**	Cytotoxic activity	Checkpoint inhibitors
**CD8+ TRM Cells**	Tissue-resident memory T cells	Localized therapies
**NK Cells**	Anti-tumor cytotoxicity	NK cell-targeted therapies
**CD4+ Th1/2/17 Cells**	Helper T cell functions	Immunomodulators
**CD4+ Tregs**	Immunosuppression	Treg inhibitors
**CD4+ Tfh T Cells**	Follicular helper functions	Immune response modulators
**Tumor-Infiltrating B Cells**	Antibody production	B cell-targeted therapies
**TAMs**	Tumor growth, angiogenesis	TAM-targeted therapies

**Table 3:** “Summary of Phase 3 Clinical Trials Targeting the Breast Cancer Microenvironment.” This table presents a comprehensive summary of phase 3 clinical trials that have specifically targeted the BC microenvironment. It highlights key findings and outcomes, providing readers with valuable insights into the status and therapeutic implications of microenvironment-targeted therapies for BC. (BC: breast cancer, TNBC: triple-negative BC, N/A: not applicable).

Number of Clinical Trial [References]	Targeted Therapy	Study Phase	Patient Cohort	Key Findings and Outcomes
**NCT03726879** **[194]**	Combining standard of care (pertuzumab-trastuzumab [PH], chemotherapy) with cancer immunotherapy	Phase 3	HER2+ BC	Did not increase pCR rates versus placebo in the ITT or PD-L1-positive populations.
**NCT00433511** **[195]**	Effect of bevacizumab	Phase 3	HER2- BC	Incorporation of bevacizumab into sequential anthracycline- and taxane-containing adjuvant therapy does not improve IDFS or overall survival.
**NCT05910710** **[N/A]**	Neoadjuvant pembrolizumab	Phase 3	TNBC	N/A
**NCT01234337** **[196]**	CapecitabinE in combination with SorafenIb or placebo	Phase 3	Advanced HER2- BC	Definitive PFS data for the combination of sorafenib and capecitabine in advanced HER2- BC and better characterize the benefit-to-risk profile.
**NCT01234337** **[197]**	Capecitabine with sorafenib or placebo	Phase 3	Advanced HER2- BC	The combination of sorafenib with capecitabine did not improve PFS, OS, or ORR in patients with HER2-negative advanced BC.
**NCT05912062** **[198]**	BC treated with neoadjuvant HP and paclitaxel	Phase 3	Early HER2+	Not only offered different biological information but importantly served as a better predictor of pCR than baseline transcriptional analysis.
**NCT00174655** **[199]**	Lymphocytic infiltration	Phase 3	node-positive, ER-negative/HER2-negative BC	Increasing lymphocytic infiltration was associated with excellent prognosis.
**NCT00004125/** **NCT00003519** **[200]**	Stromal lymphocytic infiltration	Phase 3	TNBC	Stromal lymphocytic infiltration constitutes a robust prognostic factor in TNBCs.
**NCT00567554** **[201]**	BRCA1/2 Mutations and Bevacizumab	Phase 3	TNBC	Bevacizumab may increase the pCR after standard neoadjuvant chemotherapy for patients with TNBC with BRCA1/2 mutations.
**NCT00567554** **[202]**	Neoadjuvant Chemotherapy with Trastuzumab or Lapatinib	Phase 3	HER2+	Prolonged anti-HER2 treatment—neoadjuvant lapatinib for 6 months, followed by adjuvant trastuzumab for 12 months—significantly improved survival compared with anti-HER2 treatment with trastuzumab alone.
**NCT00543127** **[203]**	Fulvestrant (Faslodex) + Anastrozole (Arimidex) vs. Anastrozole	Phase 3	HER2+/HER2-	Statistically significant increase in DFS by adding adjuvant Fulvestrant to Anastrozole, though no firm conclusions can be drawn because of the limited sample size due to the early stop of the trial.
**NCT03373708** **[N/A]**	Chemotherapy and Intensive Endocrine Therapy	Phase 2/3	Luminal B1	N/A
**NCT03580395** **[N/A]**	Apatinib, paclitaxel, cisplatin	Phase 2/3	TNBC, HER2+ or Luminal B	N/A
